# Head-to-head evaluation of host blood biomarkers for point-of-care TB triage: diagnostic accuracy study

**DOI:** 10.1128/jcm.00046-26

**Published:** 2026-07-27

**Authors:** Kevin Komakech, Ivan Sserwadda, James Sserubiri, Moses Joloba, Grant Theron, Frank Cobelens, Willy Ssengooba

**Affiliations:** 1 Department of Medical Microbiology, School of Biomedical Sciences, College of Health Sciences, Makerere University 373384, Kampala, Uganda; 2 Department of Global Health, Amsterdam Institute for Global Health and Development, Amsterdam University Medical Centres 522567, Amsterdam, Netherlands; 3 Department of Immunology and Molecular Biology, School of Biomedical Sciences, College of Health Sciences, Makerere University 373384, Kampala, Uganda; 4 National Tuberculosis Reference Laboratory/WHO Supranational Reference Laboratory, Kampala, Uganda; 5 Department of Science and Technology-National Research Foundation Centre of Excellence for Biomedical Tuberculosis Research, South African Medical Research Council Centre for Tuberculosis Research, Division of Molecular Biology and Human Genetics, Faculty of Medicine and Health Sciences, Stellenbosch University 121470, Cape Town, South Africa; 6 Makerere University Lung Institute 686538, Kampala, Uganda; University of Western Australia, Perth, Australia

**Keywords:** diagnostic accuracy study, host blood protein biomarkers, point-of-care TB triage, tuberculosis

## Abstract

Global tuberculosis (TB) incidence is slowly decreasing, partly because undiagnosed TB continues to fuel transmission. Point-of-care TB triage tests (TTTs) performed near a patient or at the site of patient care may help bridge this gap, but head-to-head comparisons of different host blood biomarkers within the same cohort are limited. In ambulatory adults in Uganda aged ≥15 years with any cough, fever, weight loss, or night sweats, we evaluated the diagnostic accuracy of C-reactive protein (CRP), monocyte-to-lymphocyte ratio (MLR), interferon-gamma-induced protein 10 (IP-10), and hemoglobin (anemia) head-to-head using a reference standard comprised of culture and/or Xpert MTB/RIF Ultra. Cut-offs of CRP and anemia were from World Health Organization (WHO) recommendations, MLR from the literature, and IP-10 from Youden’s index. We included 623 participants (193 people living with HIV [PLWHIV]), of whom 111 (18%) were reference standard-positive. Sensitivity and specificity (95% CI) were 91% (84%–96%) and 78% (74%–81%) for CRP (≥10 mg/L); 72% (63%–78%) and 80% (77%–84%) for MLR (≥0.378); 86% (79%–92%) and 73% (69%–77%) for IP-10 (≥28.75); 69% (60%–78%) and 76% (72%–80%) for anemia (<12 g/dL [female], <13 g/dL [male]), respectively. At 90% sensitivity, specificity was 76% (58%–83%) for CRP; 62% (47%–74%) for MLR; 64% (47%–75%) for IP-10, and 28% (17%–51%) for anemia. There was no significant difference for any of the four TTTs in diagnostic odds ratio between participants with and without a history of previous TB, between smokers and non-smokers, and between males and females. However, MLR performed significantly (*P* = 0.001) worse in PLWHIV. Combining two index tests in parallel testing improved sensitivity up to 96% at the expense of reduced specificity. The applicable WHO minimum target product profile of 90% sensitivity and 60% specificity was met for CRP irrespective of HIV status, and for IP-10 and MLR when cut-offs were adjusted. Sex, prior history of smoking, and TB did not impact diagnostic performance. Parallel testing may be suitable in high TB prevalence settings, where minimizing missed cases and ensuring timely referral for confirmatory testing are priorities.

**IMPORTANCE:**

This study provides strong evidence that host blood protein biomarkers (C-reactive protein [CRP], interferon gamma-induced protein 10 [IP-10], and monocyte-to-lymphocyte ratio [MLR]) can serve as effective point-of-care tools for tuberculosis (TB) triage. CRP reliably meets the World Health Organization (WHO) high-sensitivity target product profile criteria across people living with HIV and HIV-negative populations, while optimized cut-offs enable IP-10 and MLR to achieve comparable performance, expanding feasible options where CRP is unavailable or unaffordable. The minimal influence of sex, smoking history, or prior TB supports broad, unstratified implementation. Evidence that parallel testing reduces missed TB cases is particularly relevant in high-burden settings. Clinically, these findings support integrating biomarker-based triage into routine screening to improve early case detection, although it may require further external validation to ensure cut-offs used are robust and not overly data-driven. For policymakers, the results justify incorporating biomarker-based point-of-care tests into national TB screening algorithms to accelerate diagnosis, improve referral efficiency, and reduce TB transmission.

## INTRODUCTION

Tuberculosis (TB) has returned as the world’s leading cause of death from a single infectious agent by 2023, following the end of the COVID-19 pandemic (1). More effort is needed to meet the End TB Strategy goal for 2035 of a 95% reduction in TB deaths and a 90% reduction in TB incidence (2).

A key challenge is that the number of undiagnosed and untreated TB patients remains high, fueling ongoing TB transmission, especially in resource-limited countries (3). However, despite reduced pricing, rapid TB testing on current platforms such as Xpert MTB/RIF Ultra remains relatively expensive and is generally not available at the community clinic level where TB patients present (4), which may account for delays and inefficient TB screening and diagnostic methods. Thus, the World Health Organization (WHO) has re-emphasized the need for efficient, rapid TB screening tools which narrow confirmatory molecular TB testing to those with the highest likelihood of having TB (5). These have been termed “TB triage tests (TTTs)” with a more recent term as “High sensitivity screening test” (HSST) because it involves a two-step screening algorithm (5, 6); as the latter term is not specific to the triaging use case, this article remains with “TTTs.” The WHO target product profile (TPP) specifies that TTTs should be of low cost, non-sputum-based, utilize easily accessible and minimally invasive biological specimens (7), and have a minimum of 90% sensitivity and 60% specificity (6). TTTs should be implementable as point-of-care (POC), defined as tests performed near a patient or at the site of patient care (8), enabling same-day clinical decision-making (9). While POC testing is often associated with small handheld devices used to quantify analytes, it also encompasses compact bench-top instruments with reduced size and complexity, such as small hematology analyzers, immunoassay platforms, and nucleic acid amplification devices (10). Therefore, POC testing may also be laboratory-based. TTTs may screen pathogen-derived biomarkers or host-derived biomarkers (11). The latter measures components from the patient’s immune response, including cytokines, chemokines, gene expression profiles, and cellular markers (11).

Although several host biomarkers have been evaluated for diagnostic performance, most studies have focused on single biomarkers. Wide variation has been reported in diagnostic accuracy for the same biomarker, reflecting heterogeneity in test platforms, with only a limited number of studies evaluating truly POC tests, as well as differences in study populations, methods, and diagnostic reference standards used (10). This evidence gap precludes comparison of the utility of different biomarkers for POC TB triaging in health facilities, except for CRP, which has been recommended only for people living with HIV (PLWHIV). We sought to address this gap by directly comparing four host biomarker-based POC tests: C-reactive protein (CRP), a non-specific biomarker of infection and inflammation whose levels significantly increase in participants with TB compared with those without TB (12), monocyte-to-lymphocyte ratio (MLR) whose levels increase due to upregulation of monocyte and downregulation of lymphocyte production in active TB (13), interferon gamma-induced protein 10 (IP-10) whose levels increase to differentiate active from latent TB (14), and hemoglobin whose low levels have been associated with active TB since anemia is a common sign among TB patients (15). These POC tests can be conducted near the patient or at the patient’s care site. They are simple to use with minimal training, portable, and provide test results quickly. This implies that they will quickly identify people likely to have TB, allowing same-day referral for confirmatory testing, supporting decentralized care and improving access to early diagnosis, especially where laboratory capacity is limited.

Therefore, we evaluated their diagnostic triage performance head-to-head against a reference standard of positive *M. tuberculosis* complex (MTBC) culture and/or Xpert MTB/RIF ultra in a single study among patients presenting to health facilities with TB-suggestive symptoms. We also evaluated the best-performing combination of the selected POC tests in parallel or in sequence.

## MATERIALS AND METHODS

### Study design and population

This was a sub-study from the Automated smartphone-based cough audio classification for rapid tuberculosis triage testing (Cough Audio triaGE for TB; CAGE-TB study), UNCST reference number: SS1536ES. In the CAGE-TB study, 767 ambulatory men and non-pregnant women ≥15 years of age who presented for care because of TB-suggestive symptoms were prospectively enrolled and evaluated at a single visit from 9 May 2023 to 2 August 2024 at Mulago National Referral Hospital TB treatment unit (outpatient), Kisenyi Health Centre IV, and Kawaala Health Center IV in Kampala, Uganda. TB-suggestive symptoms were defined as at least two (if HIV negative) or one (if living with HIV) of cough, fever, night sweats, or weight loss. All participants were tested for HIV infection irrespective of whether they knew their status. Participants who had received any TB treatment within the past 6 months or who did not provide written informed consent were excluded.

### Sample collection

Research assistants obtained demographic and clinical information, including TB symptoms, HIV status, history of TB treatment, diabetic status, and existence of any chronic condition, after obtaining written informed consent. Whole venous blood (3–5 mL) was collected in ethylenediaminetetraacetic acid (EDTA) tubes for complete blood count (CBC) and long-term plasma storage at −20^0^C. Two (for HIV negative) or three spot sputum samples (for PLWHIV) were collected at an interval of at least 1 hour in sterile sputum containers and placed at 2°C–80°C before TB culture and Xpert MTB/RIF Ultra tests.

### Index tests methods

Although we used platforms designed for finger-prick blood testing, limited test kit availability forced us to perform CRP and IP-10 on stored samples. CRP was quantified in plasma retrospectively using the portable POC LumiraDx CRP test platform (Roche Diagnostics, UK). The test reports CRP levels in the 5–250 mg/L range within 4 minutes of adding 20 μL of plasma. Briefly, patient details were entered into the LumiraDx instrument before inserting the LumiraDx test strip on which 20 μL of plasma was placed. The instrument later displayed test results in mg/L of CRP quantified. CRP levels ≥10 mg/L were considered positive according to the literature and the WHO recommendation (5). IP-10 was quantified in plasma retrospectively using a POC rapid semi-quantitative lateral flow assay (LFA; Mondial Diagnostics, Netherlands). Following sample analysis, the line developed on the test strip was quantified by measuring its optical density at 525 nm wavelength using a battery-powered portable cube reader (Chembio Diagnostics, Germany). Briefly, 50 μL of assay fluid was dispensed into the test tube followed by 1 μL of plasma and gently shaken to attain a uniform mixture. The IP-10 test strip was immersed in the reaction mixture and left for 20 minutes before quantifying the optical density of the test line using the reader, which provided a numeric readout in arbitrary units proportional to the IP-10 concentration in the test sample. Since no established cut-off for this assay had yet been established, the IP-10 concentration cut-off was estimated from the receiver-operator characteristic (ROC) curve using the Youden index (16). CRP and IP-10 testing commenced on 10 June 2024 and was performed by an independent technologist blinded to the reference standard results. Hemoglobin was quantified in capillary finger-prick blood in real time in the CAGE-TB study using HemoCue Hb 801 System (HemoCue AB, Ängelholm, Sweden). The device reports hemoglobin levels from 1.0 to 25.6 g/dL within 1 second of placing 10 μL of blood. Briefly, HemoCue Hb 801 Microcuvettes were filled with 10 μL of finger-prick blood before inserting into the HemoCue Hb 801 device, which displayed numeric Hb levels in g/dL. WHO criteria were used to classify Hb concentration as abnormal (anemic) if the value was ≤12 g/dL or ≤13 g/dL in females or males, respectively (17). The MLR was determined from the complete blood cell count in venous whole blood collected in EDTA tubes within 6 hours of sample collection in the CAGE-TB study using a Mindray BC-5000 5-Part Hematology Analyzer (Mindray Bio-Medical Electronics, Shenzhen, China), a POC assay that reports numeric values of monocytes and lymphocytes counts in cells per microliter. The MLR cut-off point was determined based on earlier studies (18, 19) that used POC devices for its quantification.

### Reference standard test method

Liquid TB culture using Mycobacterial Growth Indicator Tube (MGIT) and Xpert MTB/RIF Ultra assay were performed as reference standard tests at the College of American Pathologists (CAP) ISO15189 accredited Mycobacteriology BSL-3 Laboratory at the School of Biomedical Sciences, Makerere University, in accordance with the laboratory’s standard operating procedures (File S1).

### Data management

Data were entered directly or from the original paper source into a Research Electronic Data Capture (REDCap, REDCap Consortium, USA) application. Participants who did not have complete information documented, including index test results and MTB culture test results, were excluded from the analysis.

### Statistical analysis

A positive reference standard was defined by a positive MTBC culture and/or positive Xpert Ultra test. We calculated median and interquartile range (IQR) for age, frequencies, and percentages for demographic characteristics such as sex, HIV and diabetic status, history of previous TB and smoking, and presence of any underlying chronic condition. In the primary analysis, we assessed the diagnostic accuracy (sensitivity, specificity, positive predictive value, and negative predictive value) of each index test individually using preset literature-derived cut-offs for CRP, MLR, and anemia (6, 17–19), and the Youden index value for IP-10. In secondary analyses, we assessed index test sensitivity at the WHO TPP, defined specificity of 60%, and specificity at the WHO TPP defined sensitivity of 90%, and diagnostic accuracy at the Youden index for each index test. Subgroup analysis was conducted for PLWHIV and HIV negatives, for males and females, and for participants with a history of TB and smoking (defined by both current and previous). We also compared the diagnostic odds ratio (DOR) of index tests among participants who had a history of TB with those who did not, and among smokers and non-smokers using a two-sided Wald test (DOR was included as a single summary measure of test performance, combining sensitivity and specificity at specified cut-off values to facilitate comparison across index tests). In a sensitivity analysis, we furthermore assessed test performance, taking the Xpert Ultra result (including trace positives) as the reference standard. Finally, we assessed combined sensitivity and specificity at the cut-offs used in the primary analysis for combinations of two index tests, performed in parallel and in sequence. For analyses involving sequential (in-series) testing, all possible testing orders were evaluated. However, because the same positivity rule was applied regardless of the testing order, the diagnostic accuracy estimates remained unchanged, as the same participants were ultimately classified as positive or negative. Therefore, the order of testing did not influence the results; rather, the diagnostic performance depended on the combination of index tests evaluated and the positivity rule applied in the sequential testing approach. All estimates were reported with a 95% confidence interval (CI) assessed by the Clopper-Pearson method.

## RESULTS

**Fig 1:**
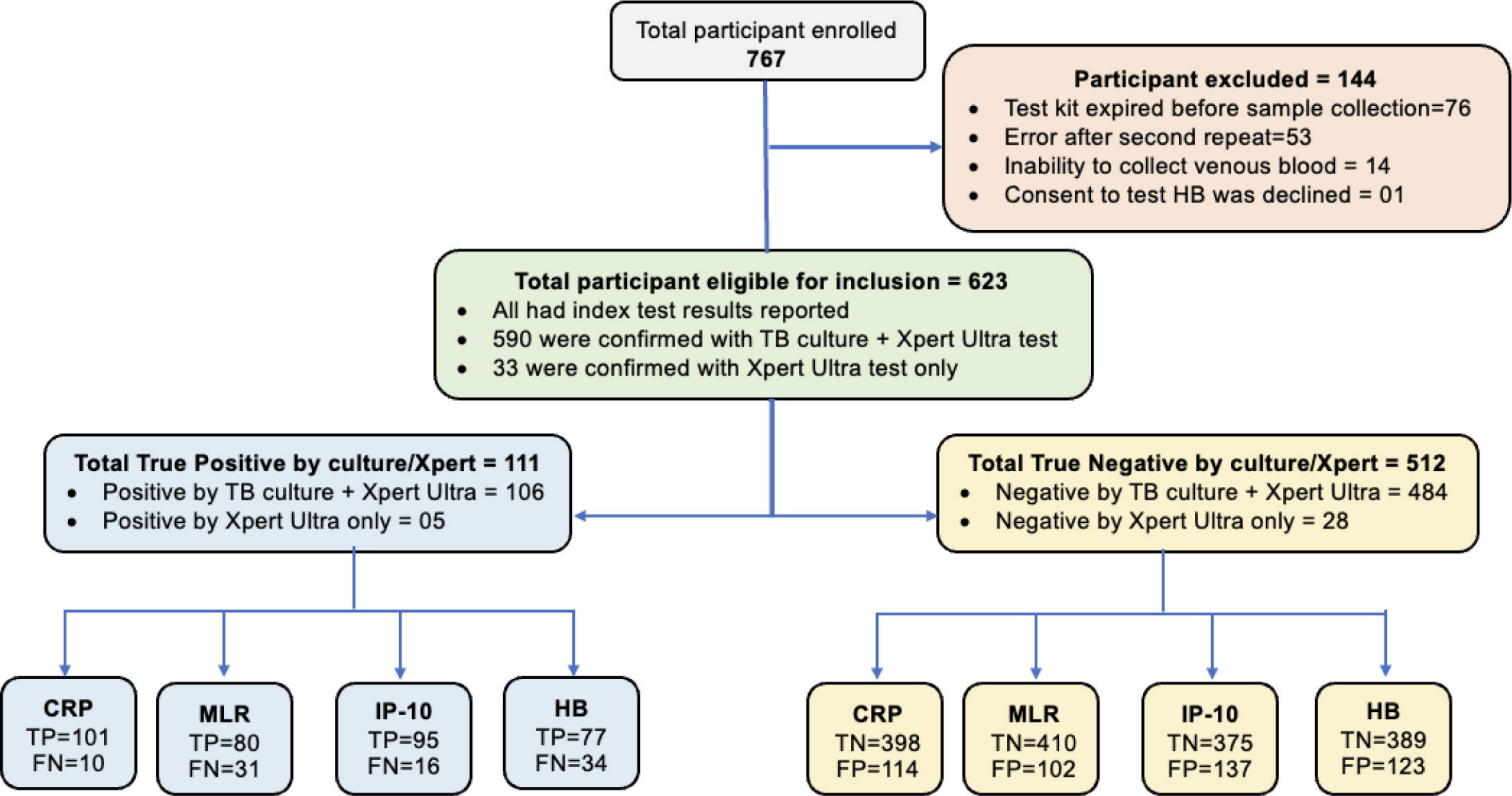
STARD diagram for flow of study participants. CRP, C-reactive protein; MLR, monocyte-to-lymphocyte ratio; IP-10, interferon gamma-induced protein 10; HB, hemoglobin; TP, true positive; TN, true negative; FP, false positive; FN, false negative.

Of the 767 participants enrolled, 623 were eligible for analysis, of whom 111 (18%) were reference standard positive (Fig. 1). The median (IQR) age was 34 (27–44) years; 370 (59%) were male, and 193 (31%) were PLWHIV. A total of 88 (14%) reported previous TB treatment, 156 (25%) reported smoking, and 44 (7%) reported the presence of any underlying chronic condition (Table S1).

### Primary analysis

**Fig 2:**
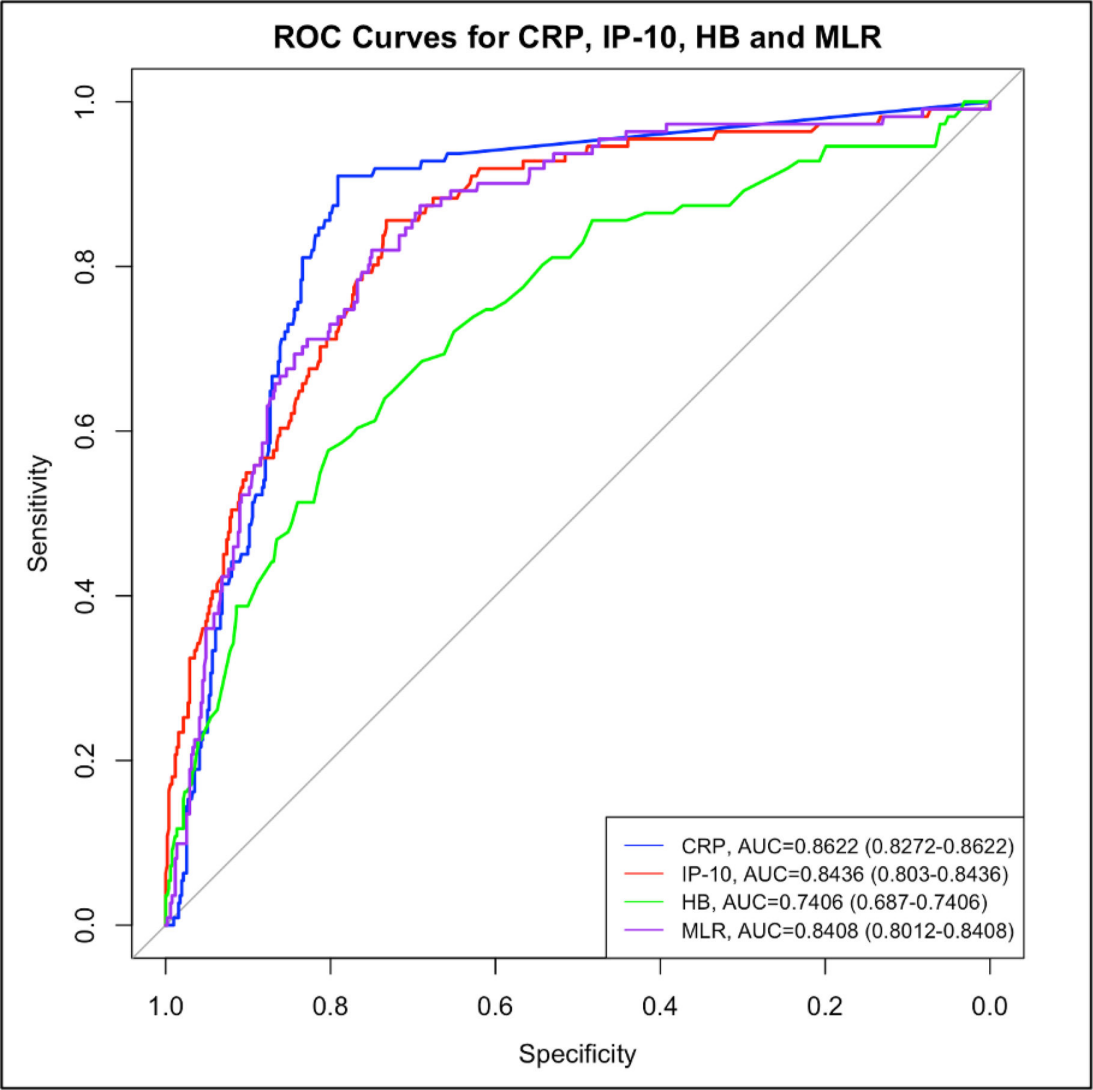
Receiver operating characteristic curve for index tests.CRP, C-reactive protein; MLR, monocyte-to-lymphocyte ratio; IP-10, interferon gamma-induced protein 10; HB, hemoglobin.

In our primary analysis, the diagnostic sensitivity and specificity (95% CI) were 91.0% (84.1%–95.6%) and 77.7% (73.9%–81.3%), respectively, for CRP at ≥10 mg/L; 72.1% (62.8%–77.8%) and 80.3% (76.6%–83.6%), respectively, for MLR at ≥0.378 cut-off; 85.6% (79.1%–92.1%) and 73.2% (69.4%–77.1%), respectively, for IP-10 at ≥28.75 cut-off; and 69.4% (59.9%–77.8%) and 76.0% (72.0%–79.6%), respectively, for anemia at cut-offs <12 g/dL for females and <13 g/dL for males (“any anemia”) (Table 1). The area under the ROC curve (AUROC; 95% CI) was highest for CRP, 86.2% (82.7%–89.7%), lowest for hemoglobin, 74.1% (68.7%–79.4%), and in between for MLR, 84.1% (80.1%–88.1%) and IP-10, 84.4% (80.3%–88.4%) (Table 2; Fig. 2). At 90% sensitivity, specificity was 78.5% (57.6%–83.4%) for CRP, 62.0% (47.1%–73.6%) for MLR, 64.3% (46.8%–74.6%) for IP-10, and 28.4% (17.2%–51.1%) for hemoglobin while at 60% specificity, the diagnostic sensitivity was 94.2% (89.8%–97.6%) for CRP, 90.1% (83.8%–95.5%) for MLR, 91.9% (85.6%–96.4%) for IP-10, and 75.2% (66.7%–83.8%) for Hb (Table 2).

**TABLE 1:** Diagnostic performance of different index tests^*a*^

Index test	Sensitivity (95% CI)	Specificity (95% CI)	PPV (95% CI)	NPV (95% CI)	Cut-off used	Reference	True positive (n/N)	True negative (n/N)
CRP	0.910 (0.841–0.956)	0.777 (0.739–0.813)	0.470 (0.402–0.539)	0.975 (0.955–0.988)	≥10 mg/L	(5)	101/111	398/512
Hemoglobin	0.694 (0.599–0.778)	0.760 (0.720–0.796)	0.385 (0.317–0.456)	0.920 (0.889–0.944)	<12 g/dL (female) < 13 g/dL (male)	(17)	77/111	389/512
MLR	0.721 (0.628–0.802)	0.803 (0.766–0.836)	0.442 (0.368–0.518)	0.930 (0.902–0.952)	≥0.378	(18)	80/111	410/512
IP-10	0.856 (0.791–0.921)	0.732 (0.694–0.771)	0.409 (0.346–0.473)	0.959 (0.939–0.979)	≥28.75)	N/A	95/111	375/512

^*a*^ CRP, C-reactive protein; MLR, monocyte-to-lymphocyte ratio; IP-10, interferon gamma-induced protein 10; CI, confidence interval; PPV, positive predictive value; NPV, negative predictive value; ELISA, enzyme-linked immunosorbent assay; AUC, area under the curve; *n*, total positives; *N*, total participants; NA, not applicable.

**TABLE 2:** AUC, specificity, and sensitivity of different index tests at 90% sensitivity and 60% specificity, respectively^*a*^

Index test	AUC (95% CI)	Cut-off at 90% sensitivity	Specificity (95% CI) at 90% sensitivity	Cut-off at 60% specificity	Sensitivity (95% CI) at 60% specificity
CRP	0.862 (0.827–0.897)	≥11.6 mg/dL	0.785 (0.576–0.834)	NA_CRP_	0.942 (0.898–0.976)
MLR	0.841 (0.801–0.881)	≥0.2727	0.620 (0.471–0.736)	≥0.2686	0.901 (0.838–0.955)
IP-10	0.844 (0.803–0.884)	≥24.1	0.643 (0.468–0.746)	≥22.5	0.919 (0.856–0.964)
Hemoglobin	0.741 (0.687–0.794)	NA_Hb_	0.284 (0.172–0.511)	NA_Hb_	0.752 (0.667–0.838)

^*a*^ CRP, C-reactive protein; MLR, monocyte-to-lymphocyte ratio; IP-10, interferon gamma-induced protein 10; AUC, area under the curve; CI, confidence interval; AUC, area under the curve; NA_Hb_, not applicable for hemoglobin (continuous data were coded binary to cater for sex cut-off difference); NA_CRP_, not applicable for C-reactive protein (at ≥5 mg/dL which was the minimum value of CRP detected, sensitivity was 100%).

### Subgroup analysis

Following sub-group analysis, index tests generally demonstrated superior diagnostic performance in the HIV-negative group compared to the PLWHIV group, with MLR showing significantly better performance in the HIV-negative (DOR: 18.1) group than in the PLWHIV (DOR: 3.4) group (*P* = 0.001) (Fig. S2; Table S2). Figure S1 further demonstrates the diagnostic performance of the index tests, all of which detected up to 45% (*n* = 50 of 111) of TB-positive participants.

When hemoglobin was further assessed based on severity, specificity gradually increased at the expense of sensitivity, i.e., sensitivity and specificity were 44.2% (32.8%–55.9%) and 39.8% (31.1%–49.1%) for mild anemia; 45.5% (34.1%–57.2%) and 62.6% (53.4%–71.2%) for moderate anemia; 10.4% (4.6%–19.4%) and 97.6% (93.0%–99.5%) for severe, respectively (Table S3).

### Sensitivity analysis

Diagnostic performance decreased when all positive Xpert Ultra results, including trace-positive results, were classified as TB positive, irrespective of culture status. That is, all biomarkers showed lower sensitivity, while specificity remained largely unchanged. Under this definition, 123 participants were classified as TB positive and 500 as TB negative by Xpert Ultra, compared with 111 TB positive and 512 TB negative participants by culture. Consequently, 12 participants were Xpert Ultra positive but culture negative and were reclassified as TB cases in the sensitivity analysis (Table S4 [overall, unstratified] and Table S5 stratified by HIV status, sex, history of tobacco smoking, and previous tuberculosis treatment].

### Combination analysis

When two index tests were used in parallel and “true positive” was defined by one or both index tests being positive and “true negative” was defined by both index tests being negative, sensitivity for the various combinations ranged from 88.3% (80.8%–93.6%) for (MLR [≥0.378] + Hemoglobin [<12 g/dL, <13 g/dL]) to 96.4% (91.0%–99.0%) for (CRP [≥10 mg/dL] + Hemoglobin [<12 g/dL, <13 g/dL]). This came at the expense of lower specificity, varying from 58.8% (54.4%–63.1%) for (IP-10 [≥0.28.75] plus Hemoglobin [<12 g/dL, <13 g/dL]) to 68.9% (64.7%–72.9%) for (CRP [≥10 mg/dL] + IP-10 [≥0.28.75]). The best combination with the highest DOR (47.0) was for (CRP [≥10 mg/dL] + MLR [≥0.378]) (Table 3). When two index tests were used sequentially and “true positive” was defined by both index tests being positive and “true negative” was defined by one or both index tests being negative, specificity for the various combinations ranged from 85.9% (82.6%–88.8%) for (CRP [≥10 mg/dL] then IP-10 [≥0.28.75]) to 92.4% (89.7%–94.5%) for (MLR [≥0.378] then Hemoglobin [<12 g/dL, <13 g/dL]) at the expense of lower sensitivity varying from 53.2% (43.4%–62.7%) for (MLR [≥0.378] then Hemoglobin [<12 g/dL, <13 g/dL]) to 81.1% (72.5%–87.9%) for (CRP [≥10 mg/dL] then IP-10 [≥0.28.75]). Here, the combination with the highest DOR (26.2) was for (CRP [≥10 mg/dL] followed by IP-10 [≥0.28.75]) (Table 4).

**TABLE 3:** Combination diagnostic accuracy when both index tests are performed simultaneously (in parallel)^*a*^

Index test	Sensitivity (95% CI)	Specificity (95% CI)	PPV (95% CI)	NPV (95% CI)	DOR (95% CI)	True positive	True negative	Subgroup
CRP (≥10 mg/L) + MLR (≥0.378)	0.955 (0.898–0.985)	0.689 (0.647–0.729)	0.400 (0.341–0.462)	0.986 (0.968–0.995)	47.0 (18.8–117.7)	106/111	353/512	“True positive” means that either of the index tests was positive “True negative” means both of the index tests were negative
CRP (≥10 mg/L) + IP10 (≥28.75)	0.955 (0.898–0.985)	0.650 (0.607–0.692)	0.372 (0.316–0.431)	0.985 (0.966–0.995)	39.4 (15.8–98.5)	90/111	333/512	
CRP (≥10 mg/L) + Hemoglobin (<12 g/dL [F], <13 g/dL [M])	0.964 (0.910–0.990)	0.631 (0.587–0.673)	0.361 (0.307–0.419)	0.988 (0.969–0.997)	45.7 (16.6–126.0)	107/111	323/512	
MLR (≥0.378) + IP-10 (≥28.75)	0.946 (0.886–0.980)	0.629 (0.585–0.671)	0.356 (0.301–0.413)	0.982 (0.961–0.993)	29.7 (12.8–68.8)	105/111	322/512	
MLR (≥0.378) + Hemoglobin (<12 g/dL [F], <13 g/dL [M])	0.883 (0.808–0.936)	0.637 (0.593–0.678)	0.345 (0.290–0.404)	0.962 (0.935–0.979)	13.2 (7.2–24.2)	98/111	326/512	
IP-10 (≥28.75) + Hemoglobin (<12 g/dL [F], <13 g/dL [M])	0.937 (0.874–0.974)	0.588 (0.544–0.631)	0.330 (0.278–0.385)	0.977 (0.954–0.991)	21.2 (9.7–46.5)	104/111	301/512	

^*a*^ CRP, C-reactive protein; MLR, monocyte-to-lymphocyte ratio; IP-10, interferon gamma-induced protein-10; CI, confidence interval.

**TABLE 4:** Combination diagnostic accuracy when both index tests are performed in sequence^*a*^

Index test	Sensitivity (95% CI)	Specificity (95% CI)	PPV (95% CI)	NPV (95% CI)	DOR (95% CI)	True positive	True negative	Subgroup
CRP (≥10 mg/L) + MLR (≥0.378)	0.676 (0.580–0.761)	0.891 (0.860–0.916)	0.573 (0.483–0.659)	0.927 (0.900–0.948)	17.0 (10.4–27.5)	75/111	444/512	“True positive” means both of the index tests were positive “True Negative” means one or both index tests were negative
CRP (≥10 mg/L) + IP-10 (≥28.75)	0.811 (0.725–0.879)	0.859 (0.826–0.888)	0.556 (0.476–0.634)	0.954 (0.931–0.972)	26.2 (15.3–44.8)	90/111	440/512	
CRP (≥10 mg/L) + Hemoglobin (<12 g/dL [F], <13 g/dL [M])	0.640 (0.543–0.729)	0.906 (0.878–0.930)	0.597 (0.503–0.686)	0.921 (0.893–0.943)	17.1 (10.5–28.0)	71/111	464/512	
MLR (≥0.378) + IP-10 (≥28.75)	0.631 (0.534–0.720)	0.906 (0.878–0.930)	0.593 (0.499–0.683)	0.919 (0.891–0.941)	16.5 (10.1–26.9)	70/111	464/512	
MLR (≥0.338) + Hemoglobin (<12 g/dL [F], <13 g/dL [M])	0.532 (0.434–0.627)	0.924 (0.897–0.945)	0.602 (0.498–0.700)	0.901 (0.872–0.925)	13.8 (8.4–22.6)	52/111	478/512	
IP-10 (≥28.75) + Hemoglobin (<12 g/dL [F], <13 g/dL [M])	0.613 (0.515–0.704)	0.904 (0.875–0.928)	0.581 (0.486–0.672)	0.915 (0.887–0.938)	14.9 (9.2–24.2)	57/111	473/512	

^*a*^ CRP, C-reactive protein; MLR, monocyte-to-lymphocyte ratio, IP-10, interferon gamma-induced protein 10; CI, confidence interval.

## DISCUSSION

Our head-to-head comparison of four POC tests showed that, among presumptive TB patients attending health facilities in Uganda, CRP performed best at a ≥10 mg/L cut-off, meeting the WHO TPP criteria for a high-sensitivity screening test: minimum sensitivity of 90% and specificity of 60%. IP-10 and MLR only met the TPP when sensitivity was fixed at 90%. Subgroup analysis revealed significant diagnostic performance in the HIV-negative group compared with the PLWHIV group. Parallel testing of the index tests improved sensitivity for various combinations at the cost of reduced specificity, with the highest diagnostic odds ratio observed for the combination of CRP (≥10 mg/dL) and MLR (≥0.378).

The search for a TB triage test to improve the feasibility and cost-effectiveness of TB diagnosis was recommended in 2014, when the WHO set TPP criteria for such a test (2). This TPP was recently updated and integrated into a generic TPP for screening tests, where a triage test is considered a “high sensitivity screening test” (6). To date, several TB triage tests, including CRP, MLR, IP-10, and hemoglobin, are either commercially available, have been evaluated, or are in the development pipeline (5). CRP, for example, has been endorsed by WHO as a high-sensitivity screening test for PTB in PLWHIV (5, 20), and several POC CRP testing platforms are now commercially available. For hemoglobin measurement only, the handheld HemoCue Hb 801 System is the most commonly used POC device (21). In contrast, MLR evaluations mostly rely on POC benchtop hematology analyzers, some of which are FDA-approved. IP-10 has usually been quantified using enzyme-linked immunosorbent assay (ELISA) (22, 23); to our knowledge, this is the first study to evaluate a lateral flow test for IP-10 (24). In our study, we used established cut-offs for CRP and hemoglobin, and published cut-offs for MLR. This cut-off has not been recommended by WHO, but previous studies using POC devices for MLR quantification employed values of 0.378 (18, 19), 0.44 (13), and 0.208 (25), while Youden’s cut-off was applied for IP-10.

With regard to each individual biomarker, earlier studies found the sensitivity and specificity of CRP for triaging TB to be below the initial WHO minimum TPP of 90% sensitivity and 70% specificity, despite using a POC device and a similar study population of presumptive TB patients with a cut-off at ≥10 mg/dL (26, 27). These differences could be attributed to variations in the sensitivity and specificity of the POC device platform and the type of sample. Decreasing specificity from 70% to 60% in the new HSST TPP played only a limited role in enabling CRP to meet the current minimum TPP, as its specificity remained up to 78%. Findings for MLR and hemoglobin fell below the initial and current WHO minimum TPP, despite the use of a POC device and similar cut-off points. These results are consistent with earlier studies that reported comparable performance (18, 19, 28). However, adjusting the cut-offs to 0.2686–0.2727 yielded 90% sensitivity and 60% specificity. Therefore, these results may be data-driven and need further confirmation in other studies. In contrast, IP-10 met the initial and current WHO minimum TPP among participants with a history of TB only. This finding contrasts with previous reports that found IP-10’s diagnostic performance to be above the initial TPP threshold irrespective of HIV status (29). The discrepancy may be due to differences in the testing method: they used a multiplex cytokine assay, which may be more sensitive than the lateral flow assay we used. However, adjusting its cut-off within the range of 22.5–24.1 achieved the current WHO minimum TPP of 90% sensitivity and 60% specificity. Therefore, this finding could also be data-driven and needs further confirmation in other studies.

There was no significant difference in the diagnostic performance of the CRP, IP-10, and hemoglobin index tests across subgroups defined by HIV status, sex, history of smoking, or history of TB (HTB). However, MLR demonstrated better performance among HIV-negative individuals compared to those who were HIV-positive individuals, although it did not meet the minimum TPP requirements. This may be attributed to the fact that HIV infection disrupts the balance of immune cells, making MLR less reflective of TB-related immune changes and more influenced by HIV-associated immune dysregulation (30, 31). In contrast, among HIV-negative individuals, MLR changes are more likely to represent TB-specific immune responses.

When Xpert Ultra, including trace-positive results, was used as the reference standard, all biomarkers showed lower sensitivity, while specificity remained largely unchanged. This reduction in sensitivity may reflect the inclusion of additional Xpert Ultra-positive/culture-negative participants who potentially had paucibacillary disease and, consequently, weaker systemic inflammatory responses that were less likely to exceed biomarker thresholds. These findings suggest that biomarker performance may vary according to disease burden, as biomarkers such as CRP, IP-10, and MLR reflect host inflammatory responses that often correlate with disease severity and bacterial burden. However, the observed differences may also reflect imperfect agreement between culture and Xpert Ultra, particularly for trace-positive results, which may represent very early disease, treated disease, residual mycobacterial DNA, or true paucibacillary TB, rather than a direct effect of bacterial load alone (32).

Combination analysis showed promising diagnostic performance, especially when the index tests were evaluated in parallel pairs. It shows that parallel testing increases diagnostic sensitivity, although it reduces specificity. This is vital when triaging TB, especially in a high-burden setting like Uganda, since missing a case (false negative) has serious public health and clinical consequences like delayed diagnosis, treatment, and continued transmission in the community (20). However, this comes at an increased cost of the TB confirmatory test, given the lower specificity observed. Nonetheless, parallel testing of TB triage biomarkers is currently in the pipeline; for example, a study by Richardson et al. (33) is validating a point-of-care multi-biomarker test (MBT) that simultaneously screens for CRP, IP-10, and serum amyloid A (SAA).

The main implication for policy following from this study is that CRP may be used to triage TB to minimize the number of tests sent for TB confirmation given current and previous evidence for its diagnostic performance from earlier studies. The use of MLR for TB triage may be restricted to HIV-negative individuals when CRP is unavailable. CRP may be used for TB triage regardless of HIV status, sex, history of previous TB, and history of smoking. If used in a TTT combination, index tests should be evaluated in parallel in pairs, and a more specific confirmatory test should follow to rule out false positives. This policy should be prioritized in areas where the incidence of TB is high. In addition, operational feasibility and decentralized applicability should be considered when interpreting the potential implementation of these TB triage tests. Although CRP and IP-10 were measured retrospectively from stored plasma samples in this study rather than under true point-of-care conditions, both assays were performed using platforms specifically designed for near-patient testing. CRP was measured using the LumiraDx CRP platform, which can operate using finger-prick blood, requires only 20 μL of sample, and provides quantitative results within approximately 4 minutes. These characteristics make it suitable for decentralized settings, although continued access to proprietary test strips and instrument maintenance would be required. IP-10 was measured using a lateral flow assay coupled to a battery-powered portable cube reader. This platform also requires a small sample volume and can operate without a conventional laboratory; however, the assay involves several manual processing steps and an incubation period of approximately 20 minutes before result interpretation, which may limit throughput in busy clinical settings. Hemoglobin was measured in real time using the HemoCue Hb 801 system, a widely used handheld device that utilizes 10 μL of capillary blood and provides results within seconds, making it highly suitable for peripheral health facilities with minimal infrastructure requirements. In contrast, MLR was derived from complete blood count measurements generated by the Mindray BC-5000 5-part hematology analyzer. Although this analyzer is available in many district and regional laboratories and can provide rapid monocyte and lymphocyte counts, its operation requires venous blood collection, a stable electricity supply, routine calibration and maintenance, quality-control procedures, and trained laboratory personnel. Consequently, MLR may be less feasible for deployment at lower-level health facilities compared with CRP, IP-10, and hemoglobin-based approaches. Future implementation studies should evaluate the real-world operational performance, costs, supply-chain requirements, and workflow integration of these platforms under routine field conditions.

Our study had limitations, including the evaluation of some index tests (CRP and IP-10) on bio-banked samples. Importantly, CRP and IP-10 were measured retrospectively from plasma stored at −80°C rather than from fresh capillary blood under true point-of-care conditions. Therefore, the diagnostic accuracy estimates reported in this study may not fully reflect performance when these biomarkers are implemented in real-time clinical settings using POC devices. This could have resulted in degradation of some biomarkers, though this was limited by storing the samples at −80°C until testing. Hence, our results may not completely reflect real-time POC testing for these assays. In addition, the study design involved a single measurement per participant, and therefore, intra-individual variability and potential diurnal fluctuations could not be assessed, which is particularly relevant for cut-offs derived from the same data set. We also performed a complete-case analysis to enable direct head-to-head comparison of all index tests within the same participant set. However, this approach may limit generalizability and introduce potential selection bias, as participants with incomplete data were excluded. The sample sizes for subgroups were also relatively small; thus, further research is recommended to further validate these findings. This was a single-center study of adult PTB patients in a high-TB-incidence setting. Therefore, the findings may not be generalizable to other populations, including children, asymptomatic individuals, patients with extrapulmonary TB, or populations in lower TB-burden settings. Additional multicenter studies across diverse epidemiological and demographic settings are needed to confirm the applicability of these findings.

In conclusion, CRP may be used to triage TB and guide referral for TB confirmation irrespective of HIV status. IP-10 and MLR met the minimum TPP after adjusting cut-offs, which requires further confirmation in other studies. When used in combination, index tests should be evaluated in parallel in pairs. If this is implemented, we may limit the number of undiagnosed and untreated TB within our community and hence progress toward achieving the End TB Strategy goal for 2035 milestones.

## Data Availability

Deidentified individual participants' data will be made available upon request to the corresponding author following institutional approval.

## SUPPLEMENTAL MATERIAL

Fig. S1 jcm.00046-26-s0001.tif Overlap of biomarkers.

Fig. S2a jcm.00046-26-s0002.tif Forest plot_By HIV.

Fig. S2b jcm.00046-26-s0003.tif Forest plot by sex.

Fig. S2c jcm.00046-26-s0004.tif Forest plot by history of smoking.

Fig. S2d jcm.00046-26-s0005.tif Forest plot by history of previous TB treatment.

Supplemental materials jcm.00046-26-s0006.docx Test procedures for TB culture and Tables S1 to S5.

Legends for Fig. S1 and S2 jcm.00046-26-s0007.docx Figure S1: Overlap of CRP (n=101), MLR (n=80), IP-10 (n=95), and HB (n=77) among TB positive participants. Figure S2: Forest plot presenting diagnostic performance following subgroup analysis by (a) HIV status; (b) Sex; (c) History of smoking; (d) History of previous TB treatment.

ASM does not own the copyrights to Supplemental Material that may be linked to, or accessed through, an article. The authors have granted ASM a non-exclusive, world-wide license to publish the Supplemental Material files. Please contact the corresponding author directly for reuse.
