# Supplementary material for: Head-to-head evaluation of host blood biomarkers for point-of-care TB triage: diagnostic accuracy study
Source: J Clin Microbiol. 2026 Jul 27;64(8):e00046-26. doi: 10.1128/jcm.00046-26 (PMC13464034; doi:10.1128/jcm.00046-26)
Supplement: Supplementary file 1 [file jcm.00046-26-s0006.docx]

**Supplementary File 1: Test procedures for TB culture**

Briefly, the first two sputum samples were homogenized, then decontaminated with Sodium Hydroxide (NaOH) (6%) – Sodium Citrate (2.9%) solution for 15 minutes in a ratio of 1:1 for NaOH-sodium citrate and homogenized sputum, respectively. 0.5% N-Acetyl-L-Cysteine (NALC) was added for every 100mL of NaOH-sodium citrate solution as a mucolytic agent, giving a final NaOH concentration of 1.5%. Phosphate-buffered saline (PBS) at a pH of 6.8 was later added up to the 45^th^ mark of 50mL falcon tube to stop the decontamination process. The decontaminated solution was then concentrated by centrifugation at 3000g for 15 minutes, and the pellet was resuspended in 1.5mL of PBS before inoculating 0.5mL in one MGIT tube for each participant. Concentrated Xpert MTB/RIF Ultra was performed by adding 2mL of sample reagent to 1mL of the sample pellet, vortexed and allowing it to stand for 15 minutes before loading 2mL of the sample mixture into the Xpert cartridge. MGIT tubes were loaded in MGIT 960 culture (Becton Dickinson Diagnostic Systems, Sparks, NV, USA), which monitored them for growth, while Xpert cartridges were loaded in GeneXpert machine (Cepheid, Sunnyvale, USA). Mycobacterium Tuberculosis Complex (MTBC) species were confirmed by a positive Ziehl-Neelsen (ZN) smear and MPT64 antigen test – BD MGIT TBc Identification Test (Becton Dickinson Diagnostic Systems, Sparks, NV, USA**)**. Samples ZN positive but MPT64 antigen negative were considered to have growth due to Non-Tuberculous Mycobacteria (NTM). [World Health Organization (1998) Laboratory Services in Tuberculosis Control. WHO/Tuberculosis/98.258, WHO, Geneva]

**Supplementary Table 1:** **Demographic and clinical characteristics of study participants** µµ

| **Demographic characteristics** | | **Frequency (N=623)** | **Percentages**  **(%)** | **TB culture** | |
| --- | --- | --- | --- | --- | --- |
|  |  |  |  | **Negative (N=512)** | **Positive (N=111)** |
| Sex | Females | 253 | 41 | 227 (44%) | 26 (23%) |
|  | Males | 370 | 59 | 285 (56%) | 85 (77%) |
| HIV status | Negative | 430 | 69 | 349 (68) | 81 (73%) |
|  | Positive | 193 | 31 | 163 (32) | 30 (27%) |
| Diabetes status | Negative | 620 | 99.5 | 509 (99%) | 111 (100%) |
|  | Positive | 3 | 0.5 | 3 (1%) | 0 (0%) |
| History of smoking  (Included both ever and current) | No | 467 | 75 | 391 (76%) | 76 (68%) |
|  | Yes | 156 | 25 | 121 (24%) | 35 (32%) |
| Previous history of TB | No | 535 | 85.9 | 440 (86%) | 95 (86%) |
|  | Yes | 88 | 14.1 | 72 (14%) | 16 (14%) |
| Any underlying chronic condition | No | 579 | 92.9 | 472 (92%) | 107 (96%) |
|  | Yes | 44 | 7.06 | 40 (8%) | 4 (4%) |

**Supplementary Table 2: Sub-group analysis by HIV status, sex, history of tobacco smoking, and previous tuberculosis treatment**

| **Index Test** | **Cut-off used** | **Sub-group** | **True positive** | **False negative** | **True negative** | **False positive** | **Sensitivity (95% CI)** | **Specificity (95% CI)** | **DOR**  **(95% CI)** | **P-Value** |
| --- | --- | --- | --- | --- | --- | --- | --- | --- | --- | --- |
| CRP | ≥10mg/dL | HIV Positives | 27 | 3 | 122 | 41 | 0.900  (0.735-0.979) | 0.748  (0.675-0.813) | 26.8  (8-93) | 0.598 |
|  |  | HIV Negatives | 74 | 7 | 276 | 73 | 0.914  (0.830-0.965) | 0.791  (0.744-0.832) | 40.0  (18-90) |  |
| MLR | ≥0.378 | HIV Positives | 15 | 15 | 126 | 37 | 0.500  (0.313-0.687) | 0.773  (0.701-0.835) | 3.4  (2-8) | 0.001* |
|  |  | HIV Negatives | 65 | 16 | 285 | 64 | 0.802  (0.699-0.883) | 0.817  (0.772-0.856) | 18.1  (0-33) |  |
| IP-10 | ≥28.75 | HIV Positives | 25 | 5 | 114 | 9 | 0.833  (0.653-0.944) | 0.933  (0.871-0.960) | 63.3  (20-205) | 0.081 |
|  |  | HIV Negatives | 70 | 11 | 261 | 88 | 0.864  (0.770-0.930) | 0.748  (0.699-0.793) | 18.9  (10-37) |  |
| Hemoglobin | <12g/dL(F) <13g/dL(M) | HIV Positives | 23 | 7 | 107 | 56 | 0.767  (0.577-0.901) | 0.656  (0.578-0.729) | 6.3  (3-16) | 0.577 |
|  |  | HIV Negatives | 56 | 25 | 276 | 73 | 0.691  (0.579-0.790) | 0.791  (0.744-0.832) | 8.5  (5-15) |  |
| CRP | ≥10mg/dL | Males | 79 | 6 | 200 | 85 | 0.929  (0.875-0.984) | 0.716  (0.663-0.768) | 30.9  (13-74) | 0.792 |
|  |  | Females | 22 | 4 | 198 | 29 | 0.846  (0.651-0.956) | 0.872  (0.822-0.913) | 37.5  (12-117) |  |
| MLR | ≥0.378 | Males | 64 | 21 | 211 | 74 | 0.753  (0.647-0.840) | 0.740  (0.685-0.790) | 8.7  (5-15) | 0.562 |
|  |  | Females | 16 | 10 | 200 | 27 | 0.615  (0.406-0.798) | 0.881  (0.832-0.920) | 11.9  (5-29) |  |
| IP-10 | ≥28.75 | Males | 73 | 12 | 200 | 85 | 0.859  (0.647-0.840) | 0.702  (0.685-0.790) | 14.3  (7-28) | 0.696 |
|  |  | Females | 22 | 4 | 175 | 52 | 0.846  (0.651-0.956) | 0.771  (0.711-0.824) | 18.5  (6-56) |  |
| Hemoglobin | <13g/dL | Males | 60 | 25 | 217 | 68 | 0.706  (0.597-0.800) | 0.761  (0.708-0.810) | 7.7  (4-13) | 0.947 |
|  | <12g/dL | Females | 19 | 7 | 166 | 61 | 0.731  (0.522-0.884) | 0.731  (0.669-0.788) | 7.4  (3-18) |  |
| CRP | ≥10mg/dL | Have a history of SMK | 33 | 2 | 86 | 35 | 0.943  (0.808-0.993) | 0.711  (0.621-0.790) | 40.5  (9-178) | 0.825 |
|  |  | No history SMK | 68 | 8 | 312 | 79 | 0.895  (0.803-0.953) | 0.798  (0.755-0.837) | 33.5  (16-73) |  |
| MLR | ≥0.378 | Have a history of SMK | 26 | 9 | 89 | 32 | 0.743  (0.567-0.875) | 0.736  (0.648-0.812) | 8.0  (3-19) | 0.498 |
|  |  | No history SMK | 54 | 22 | 322 | 69 | 0.711  (0.595-0.809) | 0.824  (0.782-0.860) | 11.4  (7-20) |  |
| IP-10 | ≥28.75 | Have a history of SMK | 28 | 7 | 91 | 30 | 0.800  (0.631-0.916) | 0.752  (0.665-0.826) | 12.1  (5-31) | 0.418 |
|  |  | No history SMK | 67 | 9 | 284 | 107 | 0.882  (0.787-0.944) | 0.726  (0.679-0.770) | 19.8  (10-41) |  |
| Hemoglobin | <12g/dL(F) <13g/dL(M) | Have a history of SMK | 23 | 12 | 88 | 33 | 0.657  (0.477-0.809) | 0.727  (0.639-0.804) | 5.1  (2-11) | 0.298 |
|  |  | No history SMK | 56 | 20 | 295 | 96 | 0.737  (0.623-0.831) | 0.754  (0.709-0.796) | 8.6  (5-15) |  |
| CRP | ≥10mg/dL | Have a history of TB | 14 | 2 | 46 | 26 | 0.875  (0.617-0.984) | 0.639  (0.517-0.749) | 12.4  (3-59) | 0.156 |
|  |  | No history of TB | 87 | 8 | 352 | 88 | 0.916  (0.841-0.963) | 0.800  (0.760-0.836) | 43.5  (20-93) |  |
| MLR | ≥0.378 | Have a history of TB | 12 | 4 | 53 | 19 | 0.75  (0.476-0.927) | 0.736  (0.619-0.833) | 8.4  (2-29) | 0.691 |
|  |  | No history of TB | 68 | 27 | 358 | 82 | 0.716  (0.614-0.804) | 0.814  (0.774-0.849) | 11.0  (7-18) |  |
| IP-10 | ≥28.75 | Have a history of TB | 15 | 1 | 51 | 21 | 0.938  (0.698-0.998) | 0.708  (0.589-0.810) | 36.4  [5-294) | 0.419 |
|  |  | No history of TB | 80 | 15 | 324 | 116 | 0.842  (0.753-0.909) | 0.736  (0.693-0.777) | 14.9  (8-24) |  |
| Hemoglobin | <12g/dL(F) <13g/dL(M) | Have a history of TB | 11 | 5 | 49 | 23 | 0.688  (0.413-0.890) | 0.681  (0.560-0.786) | 4.7  (1-15) | 0.416 |
|  |  | No history of TB | 68 | 27 | 334 | 106 | 0.716  (0.614-0.804) | 0.760  (0.716-0.798) | 7.9  (5-13) |  |

**Supplementary Table 4:** **Diagnostic performance of different index tests considering Xpert Ultra as a reference standard.**

| **Index Test** | **Sensitivity (95% CI)** | **Specificity (95% CI)** | **PPV**  **(95% CI)** | **NPV**  **(95% CI)** | **Cut-off used** | **Reference** | **True positive** | **True negative** |
| --- | --- | --- | --- | --- | --- | --- | --- | --- |
| CRP | 0.821  (0.741-0.884) | 0.772  (0.732-0.808) | 0.469  (0.401-0.538) | 0.946  (0.919-0.965) | ≥10mg/L | [5] | 101/123 | 386/500 |
| Hemoglobin | 0.634  (0.542-0.719) | 0.756  (0.715-0.793) | 0.390  (0.321-0.461) | 0.893  (0.860-0.921) | <12g/dL (Female) <13g/dL (Male) | [17] | 78/123 | 378/500 |
| MLR | 0.650  (0.559-0.734) | 0.798  (0.760-0.832) | 0.441  (0.368-0.517) | 0.902  (0.871-0.928) | ≥0.378 | [18] | 80/123 | 399/500 |
| IP-10 | 0.780  (0.696-0.850) | 0.728  (0.686-0.766) | 0.413  (0.349-0.480) | 0.930  (0.901-0.954) | ≥28.75) | N/A | 96/123 | 364/500 |

**Key:** CRP – C-reactive protein, MLR – Monocyte-Lymphocyte ratio, IP-10 – Interferon gamma induced protein 10, CI – Confidence Interval, PPV – Positive predictive value, NPV – Negative predictive value, ELISA – Enzyme-linked immunosorbent assay, AUC – Area under the curve, n = Total positives, N = Total participants, NA – Not applicable.

**Supplementary Table 5: Sub-group analysis by HIV status, sex, history of tobacco smoking, and previous tuberculosis treatment considering Xpert Ultra as a reference standard.**

| **Index Test** | **Cut-off used** | **Sub-group** | **True positive** | **False negative** | **True negative** | **False positive** | **Sensitivity (95% CI)** | **Specificity (95% CI)** |
| --- | --- | --- | --- | --- | --- | --- | --- | --- |
| CRP | ≥10mg/dL | HIV Positives | 27 | 6 | 119 | 41 | 0.818  (0.645-0.930) | 0.743  (0.668-0.809) |
|  |  | HIV Negatives | 74 | 16 | 267 | 73 | 0.822  (0.727-0.895) | 0.785  (0.738-0.828) |
| MLR | ≥0.378 | HIV Positives | 16 | 17 | 124 | 36 | 0.485  (0.307-0.664) | 0.775  (0.702-0.837) |
|  |  | HIV Negatives | 64 | 26 | 275 | 65 | 0.711  (0.606-0.802) | 0.808  (0.763-0.849) |
| IP-10 | ≥28.75 | HIV Positives | 27 | 6 | 113 | 47 | 0.818  (0.645-0.930) | 0.706  (0.629-0.776) |
|  |  | HIV Negatives | 69 | 21 | 251 | 89 | 0.766  (0.665-0.849) | 0.738  (0.688-0.784) |
| Hemoglobin | <12g/dL(F) <13g/dL(M) | HIV Positives | 24 | 9 | 108 | 52 | 0.727  (0.544-0.867) | 0.675  (0.596-0.746) |
|  |  | HIV Negatives | 54 | 36 | 270 | 70 | 0.600  (0.491-0.701) | 0.794  (0.747-0.835) |
| CRP | ≥10mg/dL | Males | 80 | 16 | 190 | 84 | 0.833  (0.745-0.901) | 0.693  (0.635-0.747) |
|  |  | Females | 21 | 6 | 196 | 30 | 0.778  (0.577-0.913) | 0.867  (0.815-0.908) |
| MLR | ≥0.378 | Males | 64 | 32 | 200 | 74 | 0.666  (0.563-0.759) | 0.729  (0.673-0.781) |
|  |  | Females | 16 | 11 | 199 | 27 | 0.592  (0.387-0.776) | 0.880  (0.830-0.919) |
| IP-10 | ≥28.75 | Males | 72 | 13 | 198 | 87 | 0.847  (0.685-0.859) | 0.695  (0.638-0.751) |
|  |  | Females | 21 | 6 | 173 | 53 | 0.777  (0.577-0.913) | 0.765  (0.704-0.819) |
| Hemoglobin | <13g/dL | Males | 72 | 24 | 189 | 85 | 0.75  (0.509-0.712) | 0.690  (0.711-0.815) |
|  | <12g/dL | Females | 19 | 8 | 168 | 58 | 0.703  (0.498-0.862) | 0.743  (0.681-0.798) |
| CRP | ≥10mg/dL | Have a history of SMK | 31 | 7 | 81 | 37 | 0.815  (0.656-0.922) | 0.686  (0.594-0.768) |
|  |  | No history SMK | 70 | 15 | 305 | 77 | 0.823  (0.725-0.897) | 0.798  (0.754-0.837) |
| MLR | ≥0.378 | Have a history of SMK | 25 | 13 | 85 | 33 | 0.657  (0.486-0.803) | 0.720  (0.630-0.799) |
|  |  | No history SMK | 55 | 30 | 314 | 68 | 0.647  (0.535-0.747) | 0.821  (0.779-0.859) |
| IP-10 | ≥28.75 | Have a history of SMK | 26 | 12 | 86 | 32 | 0.684  (0.513-0.824) | 0.728  (0.639-0.806) |
|  |  | No history SMK | 70 | 15 | 278 | 104 | 0.823  (0.725-0.897) | 0.727  (0.680-0.771) |
| Hemoglobin | <12g/dL(F) <13g/dL(M) | Have a history of SMK | 20 | 18 | 85 | 33 | 0.526  (0.358-0.690) | 0.720  (0.630-0.799) |
|  |  | No history SMK | 58 | 27 | 293 | 89 | 0.682  (0.572-0.779) | 0.767  (0.721-0.808) |
| CRP | ≥10mg/dL | Have a history of TB | 15 | 5 | 43 | 26 | 0.75  (0.508-0.913) | 0.632  (0.506-0.746) |
|  |  | No history of TB | 86 | 17 | 343 | 89 | 0.834  (0.748-0.901) | 0.793  (0.752-0.831) |
| MLR | ≥0.378 | Have a history of TB | 14 | 6 | 51 | 17 | 0.70  (0.457-0.881) | 0.75  (0.630-0.847) |
|  |  | No history of TB | 66 | 37 | 348 | 84 | 0.640  (0.540-0.732) | 0.805  (0.765-0.841) |
| IP-10 | ≥28.75 | Have a history of TB | 16 | 4 | 48 | 20 | 0.8  (0.563-0.943) | 0.705  (0.582-0.810) |
|  |  | No history of TB | 80 | 23 | 316 | 116 | 0.776  (0.684-0.852) | 0.731  (0.687-0.772) |
| Hemoglobin | <12g/dL(F) <13g/dL(M) | Have a history of TB | 11 | 9 | 47 | 21 | 0.550  (0.315-0.769) | 0.691  (0.567-0.797) |
|  |  | No history of TB | 67 | 36 | 331 | 101 | 0.650  (0.550-0.741) | 0.766  (0.723-0.805) |

**Key:** CRP – C-reactive protein, MLR – Monocyte-Lymphocyte ratio, IP-10 – Interferon gamma induced protein 10, CI – Confidence Interval, PPV – Positive predictive value, NPV – Negative predictive value, ELISA – Enzyme-linked immunosorbent assay, AUC – Area under the curve, n = Total positives, N = Total participants, NA – Not applicable.

**Supplementary Table 3: Diagnostic performance of hemoglobin based on anemia grading**

| **Index Test** | **Cut-off used** | **Sensitivity (95% CI)** | **Specificity (95% CI)** | **PPV**  **(95% CI)** | **NPV**  **(95% CI)** | **True positive** | **True negative** | **Anemia severity** |
| --- | --- | --- | --- | --- | --- | --- | --- | --- |
| Hemoglobin (Anemia) | 11-11.9g/dL (Female)  11-12.9g/dL (Male) | 0.442  (0.328-0.559) | 0.398  (0.311-0.491) | 0.315  (0.229-0.411) | 0.533  (0.425-0.637) | 34/77 | 49/123 | Mild |
|  | 08-10.9g/dL  (Female & Male) | 0.455  (0.341-0.572) | 0.626  (0.534-0.712) | 0.432  (0.322-0.547) | 0.647  (0.554-0.732) | 35/77 | 77/123 | Moderate |
|  | <8g/dL  (Female & Male) | 0.104  (0.046-0.194) | 0.976  (0.930-0.995) | 0.727  (0.390-0.940) | 0.635  (0.562-0.704) | 8/77 | 120/123 | Severe |

CI – Confidence Interval, PPV – Positive predictive value, NPV – Negative predictive value

**Supplementary Figure 1: Overlap of CRP (n=101), MLR (n=80), IP-10 (n=95) and HB (n=77) among TB positive participants**

**Key:** CRP – C-reactive protein, MLR – Monocyte-Lymphocyte ratio, IP10 – Interferon gamma induced protein 10, HB – Hemoglobin
