# Supplementary material for: Head-to-head evaluation of host blood biomarkers for point-of-care TB triage: diagnostic accuracy study
Source: J Clin Microbiol. 2026 Jul 27;64(8):e00046-26. doi: 10.1128/jcm.00046-26 (PMC13464034; doi:10.1128/jcm.00046-26)
Supplement: Supplementary file 2 [file jcm.00046-26-s0007.docx]

Supplemental legends

**Supplementary Figure 1: Overlap of CRP (n=101), MLR (n=80), IP-10 (n=95) and HB (n=77) among TB positive participants**

**Key:** CRP – C-reactive protein, MLR – Monocyte-Lymphocyte ratio, IP10 – Interferon gamma induced protein 10, HB – Hemoglobin

**Supplementary Figure 2: Forest plot presenting diagnostic performance following subgroup analysis by (a) HIV status; (b) Sex; (c) History of smoking; (d) History of previous TB treatment**

**Key:** CRP – C-reactive protein, MLR – Monocyte-Lymphocyte ratio, IP10 – Interferon gamma induced protein 10, HB – Hemoglobin
